# Traction force microscopy using temporal information

**DOI:** 10.1371/journal.pcbi.1014855

**Published:** 2026-11-02

**Authors:** Kazuko Hamaoka, Kasumi Kusakari, Hirokazu Tanimoto

**Affiliations:** Department of Science, Yokohama City University, Yokohama-City, Kanagawa, JAPAN; Northeastern University, UNITED STATES OF AMERICA

## Abstract

Traction force microscopy is a simple, non-invasive method that can quantify cellular contact forces at the cell-substrate interface. It solves an inverse problem of determining the force field from measured substrate deformation. Because this problem is ill-posed with a large condition number, it is necessary to suppress the amplification of measurement noise during inversion. Previous studies have employed algorithms such as regularization or filtering that utilize spatial information to discriminate noise and true signals. Here, we propose an alternative approach that utilizes temporal information of the system. This method employs Kalman filter, a simple data assimilation algorithm in time series analysis, to recover the force field. Using numerically generated data, we show that the method is more accurate than standard algorithms including L2-regularization and is effective at filtering out temporal noise. Using experimental data on locomoting cells, we demonstrate that the method can reliably infer time-varying cellular traction forces. We also demonstrate two extensions of the proposed method: incorporation of additional knowledge and direct inference of low-dimensional force characteristics. These practical advantages will motivate the use of proposed framework for analyzing dynamic cell mechanics.

## Author summary

Traction force microscopy (TFM) is a common experimental method to determine the interfacial forces at the boundary of cells and their environment. TFM involves a delicate computational process that solves an ill-posed inverse problem to determine the interfacial forces from measured substrate deformation. Whereas available force-recovery algorithms predominantly rely on spatial information on the system, cells are dynamic, and their dynamics must contain temporal information, which has rarely been utilized in the traction force recovery problem. In this study, we propose an approach that utilizes temporal information on the system. This method employs Kalman filter, a simple data assimilation algorithm in time series analysis, to recover the force field. Using synthetic and experimental data, we show that the method is more accurate than standard algorithms including L2-regularization and can reliably infer time-varying cellular traction forces. We also demonstrate two extensions of the proposed method: incorporation of additional knowledge as a prior and direct inference of low-dimensional force characteristics.

## Introduction

Cells exert contact forces on their surroundings. Contractile forces of adherent cells, or traction forces, are probably the most prominent and well-characterized cellular contact forces [1–3]. Adherent cells generate traction forces through the actomyosin complex and utilize these forces to deform and propel themselves as well as to probe their mechanical environment. Other types of cellular contact forces also exist; examples include the extensile forces of motile amoeba slugs [4] and the combination of pushing and pulling forces during phagocytic engulfment [5].

Traction force microscopy (TFM) is a method for quantitatively determining the contact forces, including traction forces, acting on the interface between cells and the environment [6–10]. In standard two-dimensional (2D) TFM, one cultures a cell on a linear elastic planar substrate and measures the in-plane deformation of the substrate surface. The 2D TFM then computes the surface force field corresponding to the observed substrate deformation by solving force balance equation. Because the adopted substrate is linearly elastic, the substrate displacement field $\left(u_{x},u_{y}\right)$ and the cellular force field $\left(T_{x},T_{y}\right)$ can generally be related as


$$\begin{array}{c} u_{x}(\vec{r})=\iint \left\{{𝒢}_{11}(\vec{r}-{\vec{r}}^{\prime })T_{x}({\vec{r}}^{\prime })+{𝒢}_{12}(\vec{r}-{\vec{r}}^{\prime })T_{y}({\vec{r}}^{\prime })\right\}dx^{\prime }dy^{\prime }, \end{array}$$ (1)

$$\begin{array}{c} u_{y}(\vec{r})=\iint \left\{{𝒢}_{21}(\vec{r}-{\vec{r}}^{\prime })T_{x}({\vec{r}}^{\prime })+{𝒢}_{22}(\vec{r}-{\vec{r}}^{\prime })T_{y}({\vec{r}}^{\prime })\right\}dx^{\prime }dy^{\prime }. \end{array}$$ (2)

Here, $\vec{r}=(x,y{)}^{𝖳}$ and ${\vec{r}}^{\prime }=(x^{\prime },y^{\prime }{)}^{𝖳}$ are 2D coordinates. For the half-space substrate considered throughout this study, Green’s function 𝒢 is given by the Boussinesq solution and takes the following form [11]:


$$\begin{array}{c} {𝒢}_{11}(\vec{r})=\frac{2x^{2}+y^{2}}{\tilde{E}r^{3}},\;{𝒢}_{12}(\vec{r})={𝒢}_{21}(\vec{r})=\frac{xy}{\tilde{E}r^{3}},\;{𝒢}_{22}(\vec{r})=\frac{x^{2}+2y^{2}}{\tilde{E}r^{3}}, \end{array}$$ (3)

where $r=|\vec{r}|$ and $\tilde{E}=\frac{4\pi E}{3}$. *E* is Young’s modulus of the substrate, and Poisson’s ratio of the substrate is set to 0.5. Because 𝒢 scales with 1/*r*, converting *T* to *u* can be seen as a smoothing or low-pass filtering process in spatial frequency. Conversely, determining *T* from *u* is a high-pass filtering process that amplifies high spatial frequency components. Because high spatial frequency components of the observed displacement field include a significant amount of measurement noise, the inversion can easily become unstable.

In typical TFM, one measures the substrate displacement at several positions and seeks to determine the cellular forces at the same positions. These displacement and force fields can be expressed as single vectors as


$$\begin{array}{c} \vec{u}=(\begin{array}{c} u_{x}({\vec{r}}_{1})\\ \vdots \\ u_{x}({\vec{r}}_{N})\\ u_{y}({\vec{r}}_{1})\\ \vdots \\ u_{y}({\vec{r}}_{N}) \end{array}),\;\vec{T}=(\begin{array}{c} T_{x}({\vec{r}}_{1})\\ \vdots \\ T_{x}({\vec{r}}_{N})\\ T_{y}({\vec{r}}_{1})\\ \vdots \\ T_{y}({\vec{r}}_{N}) \end{array}), \end{array}$$ (4)

where *N* denotes the number of sampled positions. $\vec{u}$ and $\vec{T}$ are related as


$$\begin{array}{c} \vec{u}=𝐆\vec{T}. \end{array}$$ (5)

**G** is discretized Boussinesq solution 𝒢 and defined as


$$\begin{array}{c} 𝐆=(\begin{array}{cc} {𝐆}_{11} & {𝐆}_{12}\\ {𝐆}_{21} & {𝐆}_{22} \end{array}), \end{array}$$ (6)

where


$$\begin{array}{c} {𝐆}_{11}(i,j)={𝒢}_{11}({\vec{r}}_{i}-{\vec{r}}_{j}),\;{𝐆}_{12}(i,j)={𝐆}_{21}(i,j)={𝒢}_{12}({\vec{r}}_{i}-{\vec{r}}_{j}),\;{𝐆}_{22}(i,j)={𝒢}_{22}({\vec{r}}_{i}-{\vec{r}}_{j}). \end{array}$$ (7)

To illustrate the problem intuitively, we consider a 1D version of the problem for a while. In this simplified problem, $T_{y}=0$ everywhere, and $T_{x}$ has a finite value only along *y* = 0 (Fig 1a and 1b). Because of symmetry, $u_{y}(x,0)=0$ regardless of $T_{x}(x,0)$ thus it is sufficient to measure $u_{x}(x,0)$ to determine $T_{x}(x,0)$. We consider a regular grid on the x-axis and denote the positions of interest using $x_{i}$ (i={1,⋯N}). The reduced displacement and force fields can be expressed as


$$\begin{array}{c} {\vec{u}}_{1D}=(\begin{array}{c} u_{x}(x_{1},0)\\ \vdots \\ u_{x}(x_{N},0) \end{array}),\;{\vec{T}}_{1D}=(\begin{array}{c} T_{x}(x_{1},0)\\ \vdots \\ T_{x}(x_{N},0) \end{array}), \end{array}$$ (8)

**Fig 1:**
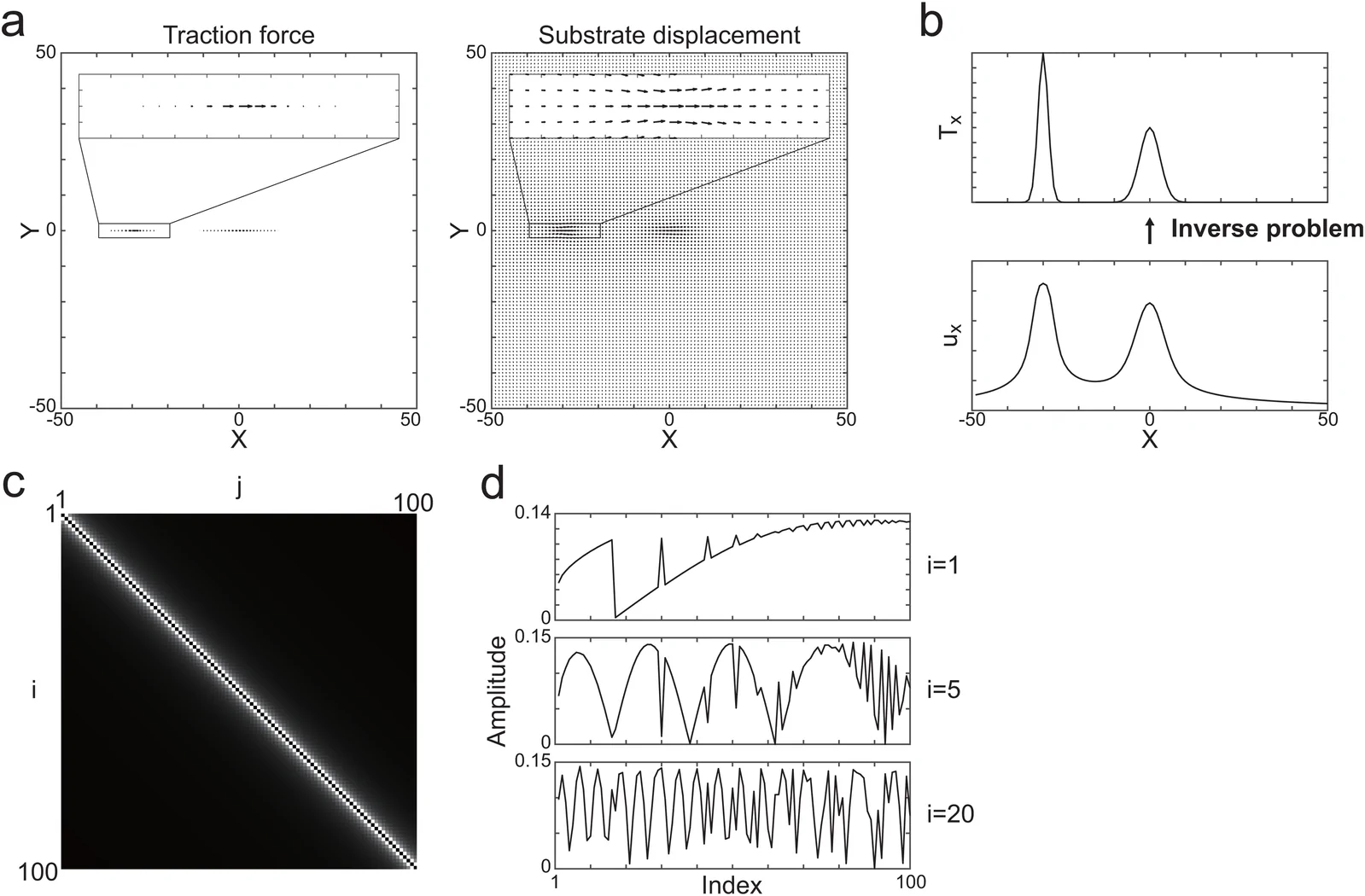
Inverse problem in TFM. **(a and b)** One-dimensional (1D) version of the force recovery problem. **(a)** $T_{x}$ distributed only on x-axis (left) was defined manually, and the corresponding substrate displacement field (right) was computed using eq. (5). **(b)** In this configuration, $T_{x}(x,0)$ (top) can be fully determined from $u_{x}(x,0)$ (bottom). **(c)** Reduced Green’s function ${𝐆}_{1D}(i,j)$ for N = 100. (d) 1st, 5th, and 20th singular vectors corresponding to the reduced Green’s function.

which are connected through the reduced Green’s function ${𝐆}_{1D}$ as


$$\begin{array}{cc} {\vec{u}}_{1D} & ={𝐆}_{1D}{\vec{T}}_{1D}, \end{array}$$ (9)

$$\begin{array}{cc} {𝐆}_{1D}(i,j) & =\frac{2}{\tilde{E}}\frac{1}{\left|x_{i}-x_{j}\right|}. \end{array}$$ (10)

In Fig 1c, ${𝐆}_{1D}$ for *N* = 100 is shown. Being a symmetric matrix, ${𝐆}_{1D}$ can be uniquely decomposed as


$$\begin{array}{c} {𝐆}_{1D}=𝐔\Sigma U^{T}=\sum_{i=1}^{N}\sigma_{i}{\vec{U}}_{i}{\vec{U}}_{i}^{T}. \end{array}$$ (11)

$𝐔=\left({\vec{U}}_{1},{\vec{U}}_{2},\cdots ,{\vec{U}}_{N}\right)$ is an orthogonal matrix, and Σ is a non-negative diagonal matrix whose diagonal elements $\sigma_{i}:=\Sigma (i.i)$ satisfy $\sigma_{1}\geq \sigma_{2}\geq \cdots \geq \sigma_{N}\geq 0$ [12]. This is a special case of singular value decomposition (SVD) where the left and right singular matrices are identical, and we call ${\vec{U}}_{i}$ and $\sigma_{i}$ the singular vector and singular value, respectively. Fig 1d shows the 1st, 5th, and 20th singular vectors. Singular vectors with larger indexes show more oscillations, indicating that they correspond to higher spatial frequency components of the displacement and force fields. The force field can be expressed in terms of **U** and Σ as


$$\begin{array}{c} {\vec{T}}_{1D}=𝐔\Sigma^{-1}U^{T}{\vec{u}}_{1D}=\sum_{i=1}^{N}\frac{{\vec{U}}_{i}^{T}{\vec{u}}_{1D}}{\sigma_{i}}{\vec{U}}_{i}. \end{array}$$ (12)

The above equation quantitatively shows the high-pass filtering characteristics of the problem: because the *i*th component in SVD is multiplied by $1/\sigma_{i}$ during the computation, higher components corresponding to smaller $\sigma_{i}$ are amplified more than the lower components. In particular, the highest component is amplified $\sigma_{1}/\sigma_{N}$ times more than the lowest component. The ratio $\sigma_{1}/\sigma_{N}$ characterizes the degree of high-pass filtering of the problem and is called condition number. The condition number of the simplified 1D problem with N = 100 is approximately $3\times 10^{3}$, indicating that the problem is an ill-conditioned inverse problem that is highly sensitive to measurement noise.

Many algorithms have been proposed to solve the inverse problem in TFM. Among them, L2-regularization, also known as Tikhonov regularization, has served as the most commonly used option since its introduction [13,14] and is actively implemented in the latest software shared in the community [15–18]. In L2-regularization, $\vec{T}$ is determined by minimizing $E=|𝐆\vec{T}-\vec{u}{|}^{2}+\lambda |\vec{T}{|}^{2}$ [19]. The first term of *E* corresponds to the observation, and the second term suppresses the artificial amplification of the force. λ is called regularization parameter and determines the balance between the two terms. $\vec{T}$ minimizing *E* has an analytical form as


$$\begin{array}{c} \vec{T}=\sum_{i=1}^{N}\frac{\sigma_{i}^{2}}{\sigma_{i}^{2}+\lambda }\frac{{\vec{U}}_{i}^{T}\vec{u}}{\sigma_{i}}{\vec{U}}_{i}. \end{array}$$ (13)

This is the naive solution (eq. (12)) multiplied by a filter factor $\sigma_{i}^{2}/(\sigma_{i}^{2}+\lambda )$, showing that L2-regularization is essentially a spatial filtering algorithm; it cuts off the high-frequency components in SVD with $\sigma_{i}\ll \sqrt{\lambda }$. Such a spatial filtering characteristic is shared by other common algorithms including L1- and other regularizations [20–22] as well as optimal filtering in the spatial frequency domain [20,23].

While existing methods predominantly rely on spatial information, cells are dynamic and continuously change their mechanical and morphological states. These time-varying processes must contain temporal information that is rarely used in the force inference. Time series analysis is a general approach to determine system state using temporal information and has been established in several fields, including mechanical engineering and economics [24,25]. Among a variety of time series analysis, Kalman filter represents a simple and versatile state-estimating algorithm [26–28]. Kalman filter is based on state-space model and provides an estimate of the system state through sequential data assimilation of dynamical measurements. Kalman filter was originally developed as an optimal state estimator for linear systems [26,29] but has also been shown to be powerful for studying non-linear systems including the mechanical behavior of living cells [30,31].

In this study, we introduce Kalman filter to solve the inversion problem in TFM. We observe that Kalman inversion outperforms standard spatial-filtering algorithms including L2-regularization. We also demonstrate two extensions of the proposed approach: incorporation of the cell shape as prior information and direct inference of low-dimensional characteristics without computing the full force field.

## Materials and methods

### Cells and substrate

Wild-type Dictyostelium discoideum (Dicty) strain AX2 cells were obtained from National BioResource Project (Tsukuba, Japan). The cells were axenically cultured as described previously [32]. The cells in exponential growth phase were diluted in balanced salts solution (BSS) buffer [33] and seeded on the substrate at a very low density (~1 cell/mm^2^) to avoid cell-cell interactions [34]. The cells were rested for 30 min in an incubator before measurements.

Commercially available silicone gel (CY 52–276; DOW Corning) was used as the flexible substrate [35,36]. The A and B solutions were thoroughly mixed at a 6:5 ratio and degassed in a vacuum desiccator. The mixed solution was then coated onto a glass-bottom dish (P50G-0–30, MatTek) using a spin coater (WS-650–23, Laurell) to obtain a substrate thickness of ~50 μm. The Young’s modulus of the substrate was approximately 1000 Pa, enough compliant to detect small traction forces of Dicty cells. Carboxylate-modified fluorescent particles with a diameter of 0.1 μm (excitation/emission 715/755 nm; F8799, Thermo Fisher Scientific) were used as fiducial markers of the substrate. To couple the fluorescent particles to the substrate, APTES (LS-3150, Shin-Etsu Chemical) was vapor-deposited onto the substrate surface to expose amino groups that bound to the particles through amine carboxyl coupling. The substrates were kept in a sealed box at room temperature and used within a week.

### Microscopy and image analysis

TFM measurements were performed using a motorized microscope (Ti2-E, Nikon) equipped with a 100x PlanApo oil-immersion objective (MRD01905, Nikon), an LED light source (X-Cite XYLIS, excelitas), and an sCMOS camera (ORCA-FusionBT, Hamamatsu). The pixel size was 0.065 μm. The equipment was controlled from Micro-Manager [37].

Time-lapse images were acquired at 1 sec interval for 1000 frames. For each time step, bright field image of the cell and fluorescent image of the fiducial marker were acquired. Cy7 filter set (ET49007, Nikon) was used for fluorescence measurements, and the same optical configuration was used for bright field imaging. The exposure time was set to be 10 ms for bright field and 250 ms for fluorescence imagings. After each time-lapse, the cells were peeled by pipetting to obtain a reference image of the substrate.

The in-plane displacement field of the substrate surface was determined by tracking individual fiducial particles using TrackMate plugin of Fiji [38,39]. The reference image was used to determine the bead positions in the undeformed state. To compensate for the small drift during measurement, average displacement of a region far from the cell at each time frame was subtracted from the displacement field at this time point. The displacement of randomly-distributed fiducial particles was converted into the displacement field of a regular grid using kriging method. The grid size was set to 16 pixels corresponding to 1.04 μm.

### Kalman inversion traction force microscopy

Kalman filter determines time-varying latent state of the system through a measurement of observables [27]. In Kalman inversion traction force microscopy (KITFM), the latent state corresponds to the cellular force field and observable corresponds to the substrate displacement field. These vector fields are encompassed into single vectors $\vec{T}$ and $\vec{u}$ (eq. (4)) and can be related as


$$\begin{array}{c} \vec{u}(k)=𝐆\vec{T}(k)+\vec{v}(k). \end{array}$$ (14)

This equation is the same as eq. (5) except for the added measurement noise $\vec{v}$. $\vec{v}(k)$ is Gaussian noise whose covariance is given by $𝐑:=\langle \vec{v}(k)\vec{v}(k^{\prime }{)}^{T}\rangle =R𝐈\delta_{kk^{\prime }}$. Discrete time is expressed using k=1,2,⋯. In the context of Kalman inversion, eq. (14) is called observation equation and the Green’s function **G** is called observation matrix. In addition to the observation equation, Kalman inversion requires a dynamic model, or state extrapolation, that describes time-evolution of the system. In standard Kalman filter adopted in this study, the general form of dynamic model is


$$\begin{array}{c} \vec{T}(k)=𝐅\vec{T}(k-1)+\vec{w}(k). \end{array}$$ (15)

**F** is a 2N×2N matrix corresponding to the time-evolution rule of the system. Because this rule is unknown, we simply set **F** = **I**. This approximation is called persistent model and has been shown to be already effective for noise filtering [24,40]. $\vec{w}$ denotes Gaussian system noise corresponding to the time-evolution of the system from k-1 to k, and its covariance is given by $𝐐:=\langle \vec{w}(k)\vec{w}(k^{\prime }{)}^{T}\rangle =Q𝐈\delta_{kk^{\prime }}$.

Given the state-space representation, Kalman filtering framework provides an estimate of the cellular force field (Fig 2) [27,41]. First, it computes the covariance of estimation error **P** and Kalman gain **K**:


$$\begin{array}{cc} {𝐏}^{-}(k) & ={𝐏}^{+}(k-1)+𝐐, \end{array}$$ (16)

$$\begin{array}{cc} 𝐊(k) & ={𝐏}^{-}(k){𝐆}^{T}{\left(𝐆{𝐏}^{-}(k){𝐆}^{T}+𝐑\right)}^{-1}. \end{array}$$ (17)

**Fig 2:**
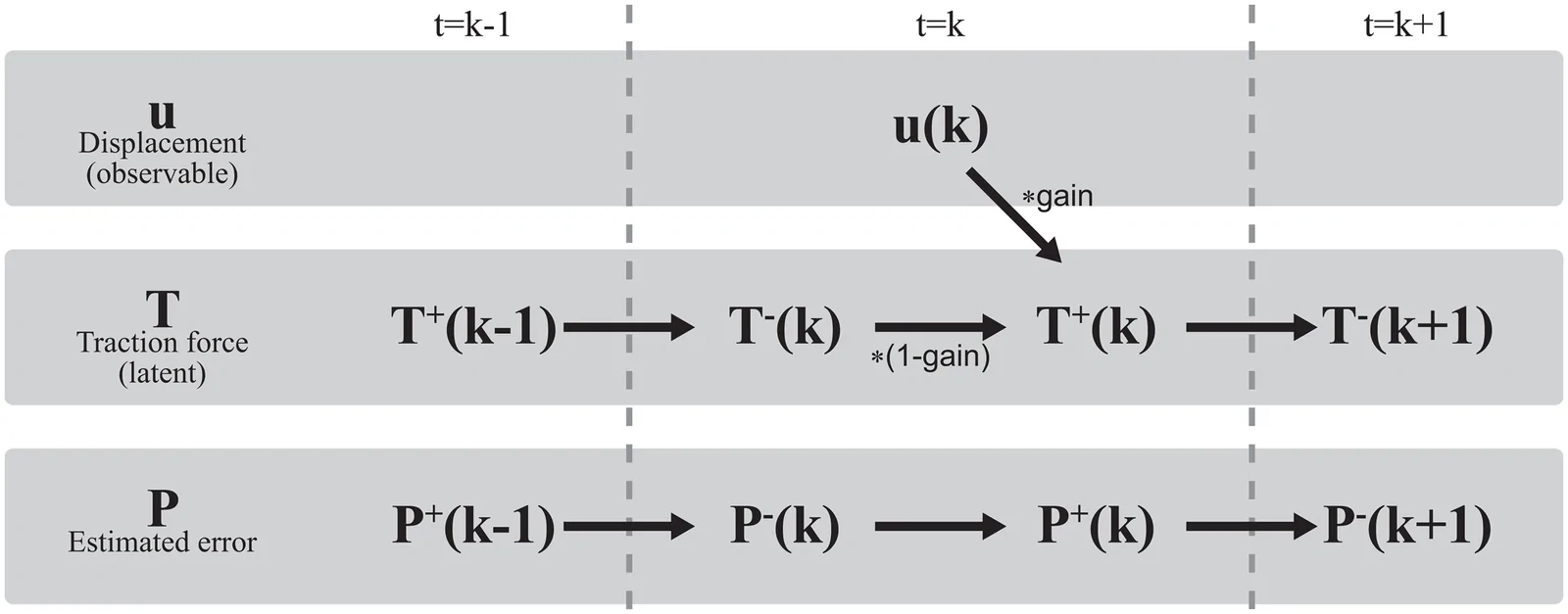
Kalman inversion traction force microscopy (KITFM). In this schematic, time is indicated with **(*k*)**, and the superscripts - (+) denote the estimates before (after) the measurement at each time step.

Superscript - (+) denotes the estimates before (after) the measurement at each time step. Because we adopt persistent model, a priori estimate of the force field before the measurement at *k*, ${\hat{\vec{T}}}^{-}(k)$, is equal to the posterior estimation at k−1:


$$\begin{array}{c} {\hat{\vec{T}}}^{\;-}(k)={\hat{\vec{T}}}^{\;+}(k-1). \end{array}$$ (18)

Subsequently, the posterior estimation of cellular forces after the measurement, ${\hat{\vec{T}}}^{+}(k)$, as well as the updated covariance of estimation error are provided by the following data fusion process:


$$\begin{array}{cc} {\hat{\vec{T}}}^{+}(k) & ={\hat{\vec{T}}}^{-}(k)+𝐊(k)\left(\vec{u}(k)-𝐆{\hat{\vec{T}}}^{-}(k)\right)\\ & =(𝐈-𝐊(k)𝐆){\hat{\vec{T}}}^{-}(k)+𝐊(k)\vec{u}(k), \end{array}$$ (19)

$$\begin{array}{cc} {𝐏}^{+}(k) & =(𝐈-𝐊(k)𝐆){𝐏}^{-}(k). \end{array}$$ (20)

The first line of eq. (19) is the standard expression of Kalman filter, whereas it is easier to see in the second line that the update is a sum of the prior estimation ${\hat{\vec{T}}}^{-}(k)$ and observation $\vec{u}(k)$ weighted by Kalman gain.

Since **R** can be determined directly by experiments, **Q** is the only free parameter in the inference. For the initial condition, we choose ${\hat{\vec{T}}}^{-}(0)=\vec{0}$ and **P**^-^(0) = *O*. Alternatively, **K**(*k*) can be replaced by its converged value to obtain mostly the same results with shorter computational time [27].

### Cell-shape constrained KITFM

KITFM framework was extended to restrict the force field within the cell. Let $i=\{n_{1},n_{2},\cdots ,n_{N^{\prime }}\}$ be the indexes that correspond to the positions outside the cell. To implement the condition $T_{x}({\vec{r}}_{i})=T_{y}({\vec{r}}_{i})=0$, the observation matrix is modified as


$$\begin{array}{cc} G_{con}(k) & =(\begin{array}{cc} 2c𝐆\\ 𝐂(k) & O\\ O & 𝐂(k) \end{array}), \end{array}$$ (21)

where **C**(*k*) is an $N^{\prime }\times N$ matrix defined as


$$\begin{array}{c} 𝐂(k)=\begin{array}{c} \begin{array}{ccccccccc} 1 & \cdots & n_{1} & \cdots & n_{2} & \cdots & n_{N^{\prime }} & \cdots & N \end{array}\\ {}[2pt]\left(\begin{array}{ccccccccc} 0 & \cdots & 1 & \cdots & 0 & \cdots & 0 & \cdots & 0\\ 0 & \cdots & 0 & \cdots & 1 & \cdots & 0 & \cdots & 0\\ \vdots & & \vdots & & \vdots & & \vdots & & \vdots \\ 0 & \cdots & 0 & \cdots & 0 & \cdots & 1 & \cdots & 0 \end{array}\right) \end{array} \end{array}$$ (22)

Accordingly, the measurement vector becomes a $2(N+N^{\prime })$ dimensional vector as


$$\begin{array}{c} {\vec{u}}_{con}(k)=(\begin{array}{c} \vec{u}(k)\\ 0\\ \vdots \\ 0 \end{array}). \end{array}$$ (23)

In addition to the original measurement $\vec{u}(k)$, ${\vec{u}}_{con}(k)$ includes additional ‘measurement’ that $T_{x}({\vec{r}}_{i})=T_{y}({\vec{r}}_{i})=0$ for the area outside the cell. To set this constraint strict, the corresponding measurement noise is set to zero as


$$\begin{array}{c} {\vec{v}}_{con}(k)=(\begin{array}{c} \vec{v}\\ 0\\ \vdots \\ 0 \end{array}),\;R_{con}(k):=\langle {\vec{v}}_{con}(k){\vec{v}}_{con}(k^{\prime }{)}^{T}\rangle =(\begin{array}{cc} 𝐑 & O\\ O & O \end{array}). \end{array}$$ (24)

Using these modified variables, the observation equation of constrained KITFM becomes


$$\begin{array}{c} {\vec{u}}_{con}(k)={𝐆}_{con}(k)\vec{T}(k)+{\vec{v}}_{con}(k). \end{array}$$ (25)

Note that the observation matrix ${𝐆}_{con}$ and measurement noise ${\vec{v}}_{con}$ are no longer stationary but change over time. By replacing $\vec{u}$, **G**, and **R** in eqs. (16–20) with the modified variables, Kalman filter algorithm obtains constrained force field using the same computational process.

### Direct inference of traction dipole

Cellular traction forces can be systematically analyzed using multipole expansion [32,42–50]. The force dipole is the lowest component of expansion and is a simple measure of cellular contractile/extensile characteristics. For 2D force field, the force dipole can be defined as


$$\begin{array}{c} D_{xx}=\iint xT_{x}(x,y)dxdy,\;D_{xy}=\iint xT_{y}(x,y)dxdy,\;D_{yx}=\iint yT_{x}(x,y)dxdy,\;D_{yy}=\iint yT_{y}(x,y)dxdy. \end{array}$$ (26)

$D_{xy}-D_{yx}$ is the net torque that a cell exerts to the substrate. Since the moment of inertia of a cell can be negligible, a cell must be torque-free thus $D_{xy}=D_{yx}$ [42]. The substrate displacement at position $\vec{r}$ induced by force dipoles at ${\vec{r}}^{\prime }$ can be expressed as [43,44]


$$\begin{array}{c} (\begin{array}{c} u_{x}(\vec{r})\\ u_{y}(\vec{r}) \end{array})=(\begin{array}{ccc} {ℋ}_{11}(\vec{r}-{\vec{r}}^{\prime }) & {ℋ}_{12}(\vec{r}-{\vec{r}}^{\prime }) & {ℋ}_{13}(\vec{r}-{\vec{r}}^{\prime })\\ {ℋ}_{21}(\vec{r}-{\vec{r}}^{\prime }) & {ℋ}_{22}(\vec{r}-{\vec{r}}^{\prime }) & {ℋ}_{23}(\vec{r}-{\vec{r}}^{\prime }) \end{array})(\begin{array}{c} D_{xx}\\ D_{xy}\\ D_{yy} \end{array}), \end{array}$$ (27)

where


$$\begin{array}{c} {ℋ}_{11}(\vec{r})=\frac{x(2x^{2}-y^{2})}{\tilde{E}r^{5}},\;{ℋ}_{12}(\vec{r})=\frac{6x^{2}y}{\tilde{E}r^{5}},\;{ℋ}_{13}(\vec{r})=\frac{x(2y^{2}-x^{2})}{\tilde{E}r^{5}},\\ {ℋ}_{21}(\vec{r})=\frac{y(2x^{2}-y^{2})}{\tilde{E}r^{5}},\;{ℋ}_{22}(\vec{r})=\frac{6y^{2}x}{\tilde{E}r^{5}},\;{ℋ}_{23}(\vec{r})=\frac{y(2y^{2}-x^{2})}{\tilde{E}r^{5}}. \end{array}$$ (28)

In direct inference of the traction dipole, the latent state is a ‘dipole vector’ $\vec{D}=(D_{xx},D_{xy},D_{yy}{)}^{T}$, and state-space model of the system reads


$$\begin{array}{cc} \vec{u}(k) & =𝐇(k)\vec{D}(k)+\vec{v}(k), \end{array}$$ (29)

$$\begin{array}{cc} \vec{D}(k) & =\vec{D}(k-1)+{\vec{w}}_{D}(k). \end{array}$$ (30)

Observation matrix **H** is given by


$$\begin{array}{c} 𝐇(k)=(\begin{array}{ccc} {ℋ}_{11}({\vec{r}}_{1}-{\vec{r}}_{c}(k)) & {ℋ}_{12}({\vec{r}}_{1}-{\vec{r}}_{c}(k)) & {ℋ}_{13}({\vec{r}}_{1}-{\vec{r}}_{c}(k))\\ & \vdots & \\ {ℋ}_{11}({\vec{r}}_{N}-{\vec{r}}_{c}(k)) & {ℋ}_{12}({\vec{r}}_{N}-{\vec{r}}_{c}(k)) & {ℋ}_{13}({\vec{r}}_{N}-{\vec{r}}_{c}(k))\\ {ℋ}_{21}({\vec{r}}_{1}-{\vec{r}}_{c}(k)) & {ℋ}_{22}({\vec{r}}_{1}-{\vec{r}}_{c}(k)) & {ℋ}_{23}({\vec{r}}_{1}-{\vec{r}}_{c}(k))\\ & \vdots & \\ {ℋ}_{21}({\vec{r}}_{N}-{\vec{r}}_{c}(k)) & {ℋ}_{22}({\vec{r}}_{N}-{\vec{r}}_{c}(k)) & {ℋ}_{23}({\vec{r}}_{N}-{\vec{r}}_{c}(k)) \end{array}). \end{array}$$ (31)

Here, ${\vec{r}}_{c}(k)$ denotes the cell centroid. By replacing **G** with **H**, $\vec{T}$ with $\vec{D}$, and **R** with ${𝐑}_{D}$ in eqs. (16–20), KITFM framework directly infers the cellular force dipole.

## Results

We first evaluated Kalman inversion traction force microscopy (KITFM) using numerically generated data. We constructed a simple dynamic pattern of localized forces that oscillated over time (Fig 3a and 3b). The amplitude and time-scale of simulated forces was chosen to obtain substrate displacements that roughly matched the cell experiments presented later. The time interval was set to be 1 sec, which is the same as the cell experiments. This time interval corresponds to the viscoelastic time-scale of typical silicone or hydrogel substrates [36], thus can be regarded as the highest temporal resolution attainable in standard TFM. The substrate displacement field corresponding to the simulated forces was computed using eq. (5), and experimentally-determined measurement noise (*R* = 1×10^–5^ μm^2^) was added to the displacement field. Even with this relatively small measurement noise, direct inversion without noise-removing processes could not recover the force field but led to a completely distorted result (Fig 3c).

**Fig 3:**
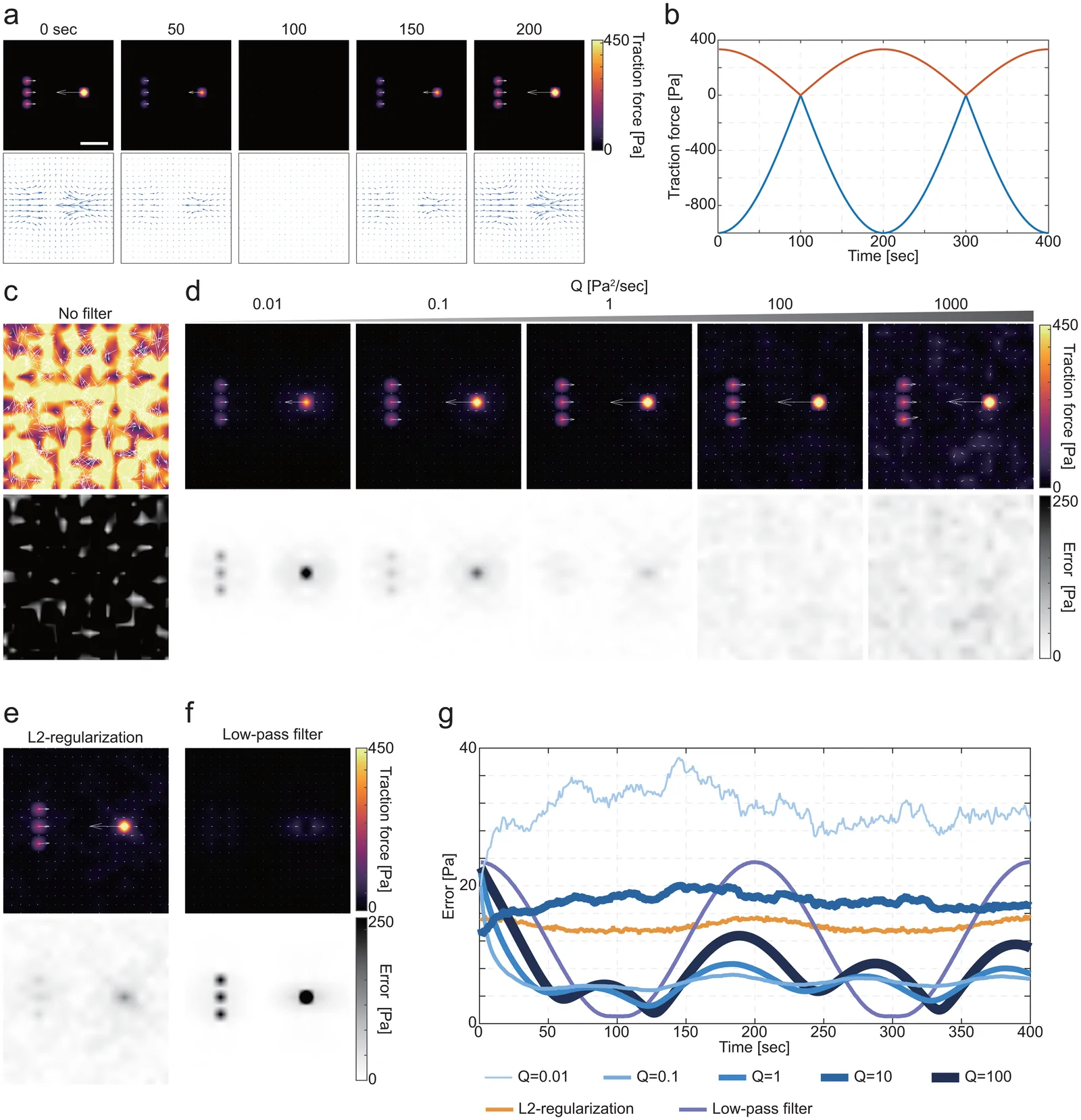
Parameter tuning and evaluation of KITFM using synthetic data. **(a and b)** Test problem. **(a)** A simple dynamic force pattern consisting of one strong and three weak force spots (top) and corresponding substrate deformation field (bottom) were numerically generated. Note that measurement noise was added to the deformation field. **(b)** Amplitude of the strong (blue) and weak (orange) force spots is plotted as a function of time. **(c-f)** Force fields obtained by different inversions. Recovered force field at t = 200 sec is shown. **(c)** Direct inversion without filtering. **(d)** KITFM with different *Q* values. **(e)** L2-regularization with l-curve criterion. **(f)** Low-pass filtering. For each result, the recovered force field (top) and its difference from the true force field (bottom) are shown. **(g)** Quantitative comparison of different inversion methods. The estimation error, defined as Root Mean Squared Error between true and inferred force fields, for each inference is plotted as a function of time. Scale bar, 5 μm.

We applied KITFM to the simulated data. Because system noise *Q* is the only free parameter of KITFM, we first assessed the effect of *Q* on the inference. For a given measurement noise, the behavior of Kalman filter is fully determined by *Q*. If *Q* is too small, the filter does not trust measurement thus cannot follow the time-varying system state properly. Conversely, if *Q* is too large, the filter trusts measurement too much and thus does not effectively filter out measurement noise. By systematically changing *Q*, we observed that KITFM could recover the force field for a range of *Q* including two orders of magnitude, ranging from 0.1 to 100 Pa^2^/sec (Fig 3d). For the best Q values (Q = 0.1-1), the estimation error was approximately 5 Pa, which is ~1% of the maximum amplitude of true forces. Given the measurement time interval of Δt = 1 sec, these *Q* values correspond to directed force evolution of $\sqrt{Q/\Delta t}\sim$0.3 to 30 Pa/sec, which is in agreement with the simulated force evolution speed of ~5 Pa/sec. These results suggest that KITFM can robustly recover the force field with moderate sensitivity to the inference parameter.

We then compared KITFM with other algorithms. Specifically, we tested two standard algorithms, L2-regularization and low-pass filtering. We observed that KITFM outperforms these standard algorithms (Fig 3e-3g). A low-pass filter with an empirical parameter failed to accurately recover the localized force patterns (Fig 3f). While L2-regularization with l-curve criterion can recover the overall force field, quantitative investigation revealed that the inference error is approximately three-fold larger than that of KITFM with the best parameter (Fig 3e and 3g). These results suggest that KITFM can infer forces more accurately than the standard algorithms.

Since KITFM utilizes temporal information, its accuracy should depend on the temporal resolution of measurement. To systematically assess the effect of temporal resolution, we changed Δt from 1 to 10 sec in the synthetic data and applied KITFM (Fig A in S1 Appendix). We observed that the estimation error became larger with increasing Δt, which is reasonable because more temporal information is lost for lower sampling frequency. The accuracy of KITFM was superior to that of L2-regularization when Δt is smaller than approximately 10 sec. These results show that the accuracy of KITFM depends on the temporal resolution, and that there is a range of adequate sampling frequency attainable in experiments.

Next, we evaluated whether KITFM was applicable for experiments. Dictyostelium discoideum (Dicty) cell is a simple unicellular model organism for studying rapid cell crawling [51]. In a weakly starved state, Dicty cells exhibit contraction/relaxation cycles with a period of several tens of seconds. We recorded the substrate displacements induced by a migrating Dicty cell in this state at 1 frame/sec for 200 sec and applied KITFM to the time-lapse data (Fig 4a-4d, Fig B in S1 Appendix). Though the initial inference condition (zero forces everywhere) deviates from the true force field, it took only a few frames for KITFM to start adhering to the data. After this initial period, the deviation became mostly stationary, suggesting that KITFM started stable inference. We also inferred traction forces from the same data using L2-regularization (Fig 4e and 4f). We observed a small spatially periodic pattern in the inferred force field (Fig 4e). This reflects that L2-regularization is essentially a filter in the spatial frequency domain. We also observed that the traction field inferred using L2-regularization exhibits noisy time evolution. In Fig 4f, the traction force at a single grid fixed in the lab-frame (+ marker in Fig 4e) is plotted as a function of time. The time-evolution contains noisy spikes. In contrast, the same analysis of the force field inferred using KITFM shows that the force field exhibits less temporal noise (Fig 4d). Importantly, KITFM can capture rapid force changes observed in L2-regularization (50–55 and 70–80 sec in Fig 4g), suggesting that KITFM filters out temporal noise while preserving fine temporal characteristics. We also confirmed that the results were mostly similar for the measurement time interval of 1–10 sec (Fig C in S1 Appendix). These results highlight the advantages of KITFM in analyzing time-varying cell tractions.

**Fig 4:**
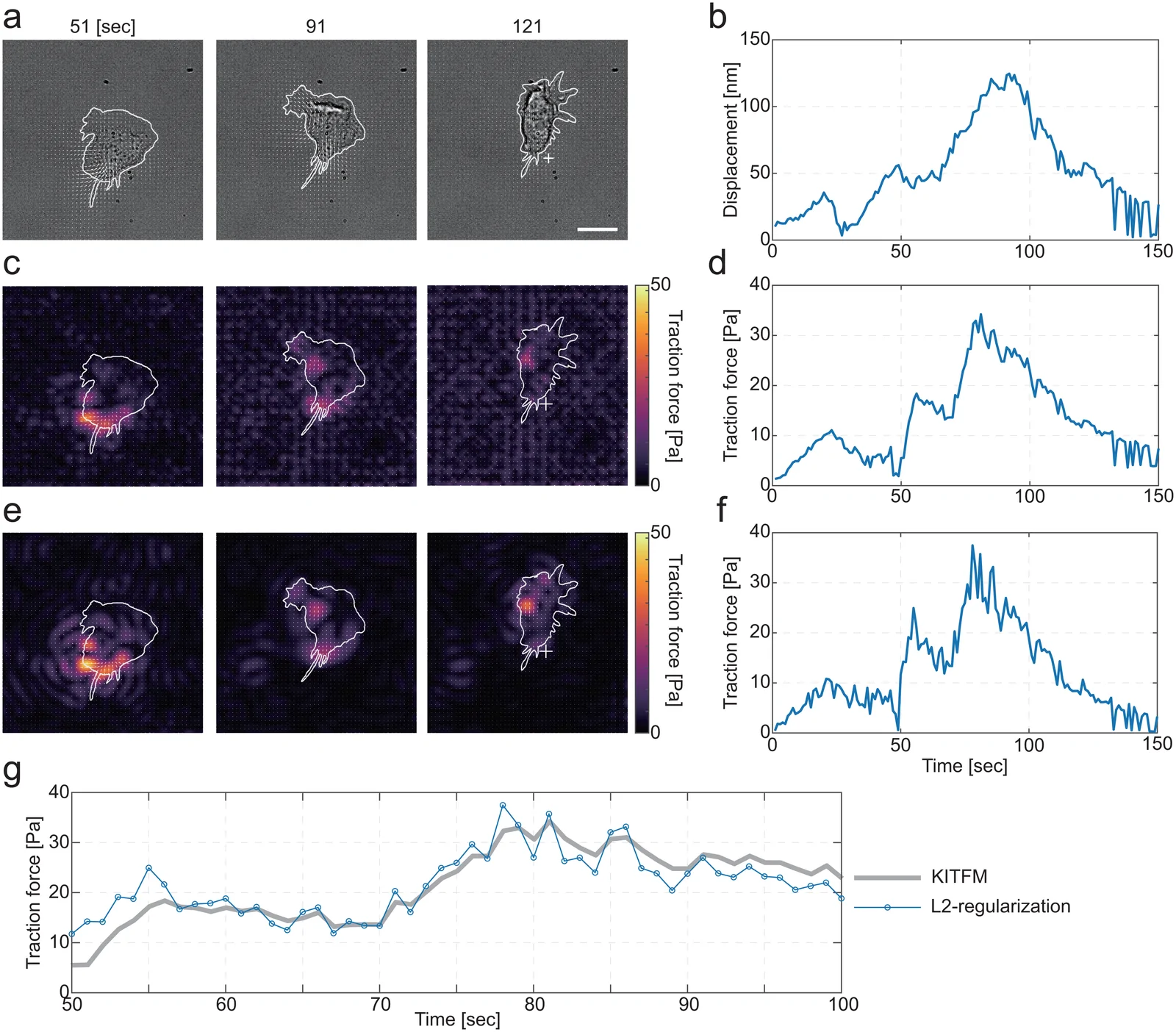
Inference of cell traction using KITFM. **(a and b)** Substrate displacement field induced by a crawling Dicty cell. **(a)** Snapshot of displacement field overlaid on bright field images. **(b)** Substrate displacement at a single grid fixed in lab-frame, shown with + marker in **(a)**, is plotted as a function of time. **(c and d)** Cellular traction forces recovered using KITFM. The traction forces were computed from the data in (a-b) using KITFM. **(c)** Snapshot of traction force field overlaid on its color-coded amplitude. **(d)** Traction force at the same grid in (b) is plotted as a function of time. **(e and f)** Cellular traction forces recovered using L2-regularization. **(g)** Enlarged plot of recovered traction force dynamics. Force evolution shown in (d) and (f) during 50-100 sec are plotted. Scale bar, 10 μm.

Lastly, we propose two extensions of KITFM. The first is the incorporation of additional information regarding the cell. As contact forces, traction forces can only be distributed at the cell-substrate interface. However, filtering process during the inversion can broaden the force field and lead to artificial traction forces outside the cell. In fact, in the force field inferred using KITFM, there are more forces outside the cell than in the force field inferred using L2-regularization (Fig 4c and 4e). This is probably because these forces are not directly penalized in KITFM, whereas they are in L2-regularization, which seeks to reduce $|\vec{T}{|}^{2}$. Taking advantage of the Bayesian nature of the inference framework, we extended KITFM to constrain the traction forces within the cell. In the cell shape-constrained KITFM, additional ‘measurement’ that specifies $T_{x}({\vec{r}}_{i})=T_{y}({\vec{r}}_{i})=0$ for the area outside the cell was implemented (Methods). The noise corresponding to these ‘measurements’ was set to be zero to make the constraint strict. With these modifications, cell-shape constrained KITFM infers traction forces that are restricted within the cell (Fig 5a and 5b). It was confirmed that the substrate displacements measured in the experiments and those corresponding to the inferred forces mostly agreed (Fig 5c and 5d). These results demonstrate that KITFM framework can be extended to implement cell-shape constraints.

**Fig 5:**
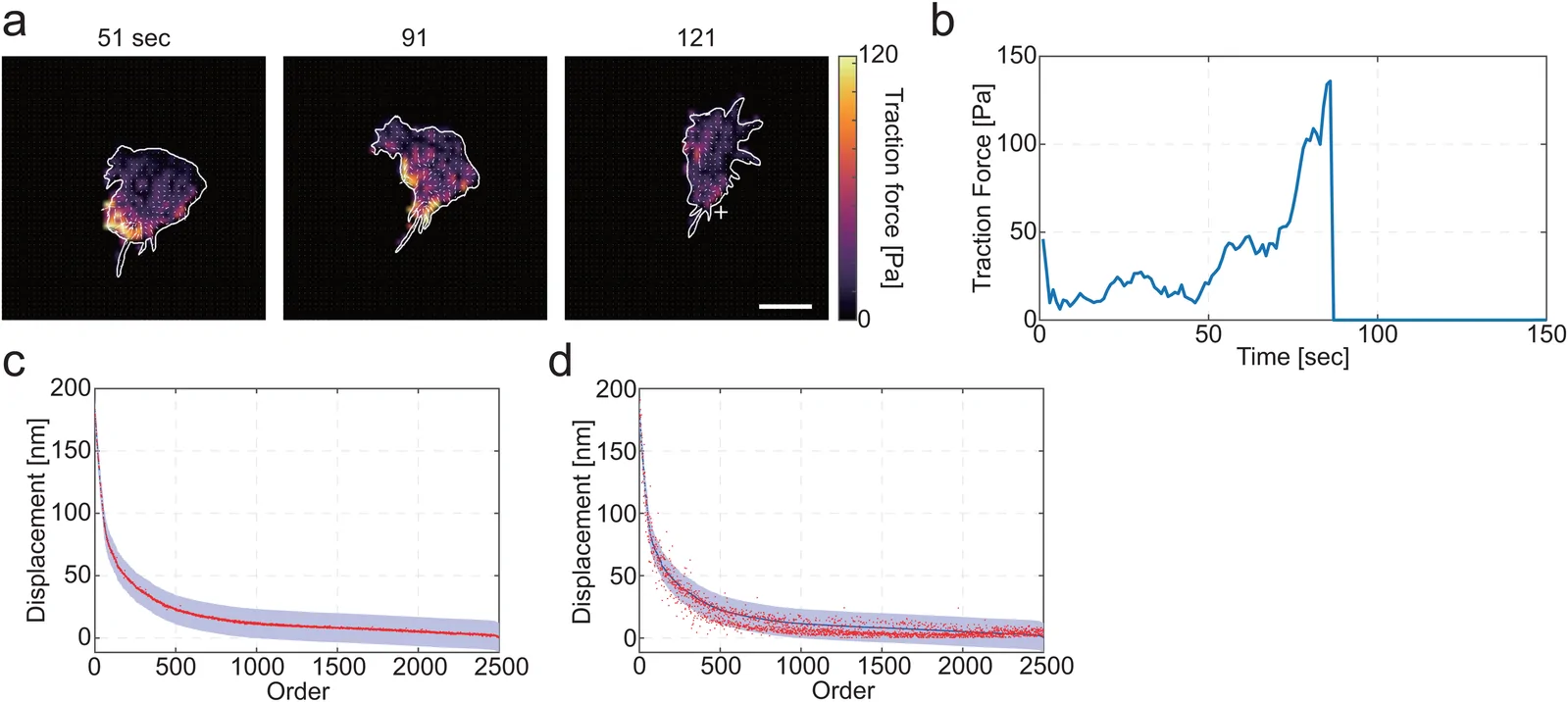
KITFM with cell-shape constraint. **(a and b)** Cellular traction forces recovered using constrained KITFM. Traction forces corresponding to the displacements presented at Fig 4a-4b were computed using cell-shape constrained KITFM. **(a)** Snapshot of traction force field overlaid on its color-coded amplitude. **(b)** Traction force at a single grid fixed in the lab-frame, shown with + marker in **(a)**, is plotted as a function of time. **(c and d)** Quantification of the inference. The degree of data fusion in original and constrained KITFM is assessed by comparing measured and recovered substrate displacements at the same grid. Blue: measured displacements ±3σ, where σ corresponds to the error of fiducial marker localization. Red: displacements corresponding to traction force field recovered using (c) original (d) or cell-shape constrained KITFM. Scale bar, 10 μm.

As a second extension, we used the KITFM framework to directly determine low-dimensional characteristics of the force field. Previous studies have shown that force multipoles are simple measures of traction force patterns such as rotational or front-rear asymmetries [32,42,44–50]. Usually, these characteristics are defined using a full force field. For instance, the force dipole, the lowest term in multipole expansion, can be defined as


$$\begin{array}{c} D_{xx}=\iint xT_{x}(x,y)dxdy,\;D_{xy}=\iint xT_{y}(x,y)dxdy,\;D_{yx}=\iint yT_{x}(x,y)dxdy,\;D_{yy}=\iint yT_{y}(x,y)dxdy. \end{array}$$ (32)

We modified KITFM to directly determine these force dipoles (Methods). In this inference, cellular latent state is expressed as a dipole vector with three components: $\vec{D}=(D_{xx},D_{xy}(=D_{yx}),D_{yy}{)}^{T}$. Note that the dimension of dipole vector is much smaller than that of the full force field $\vec{T}$, which has ~5000 degrees of freedom. With appropriately modifying observation matrix (eq. (31)), the KITFM framework can directly determine the traction force dipoles (Fig 6a). The inferred dipoles were compared with those defined using full force field (Fig 6b). The results indicate that these dipoles mostly agree well, while the two dipoles showed some deviation when the cell tractions became weak. We also analyzed the correlation between the directly inferred force dipole and cell velocity orientation to find that these two axes agreed well (Fig 6c). These results confirm previous results [44] and demonstrate that the KITFM framework can be used to directly determine low-dimensional force characteristics.

**Fig 6:**
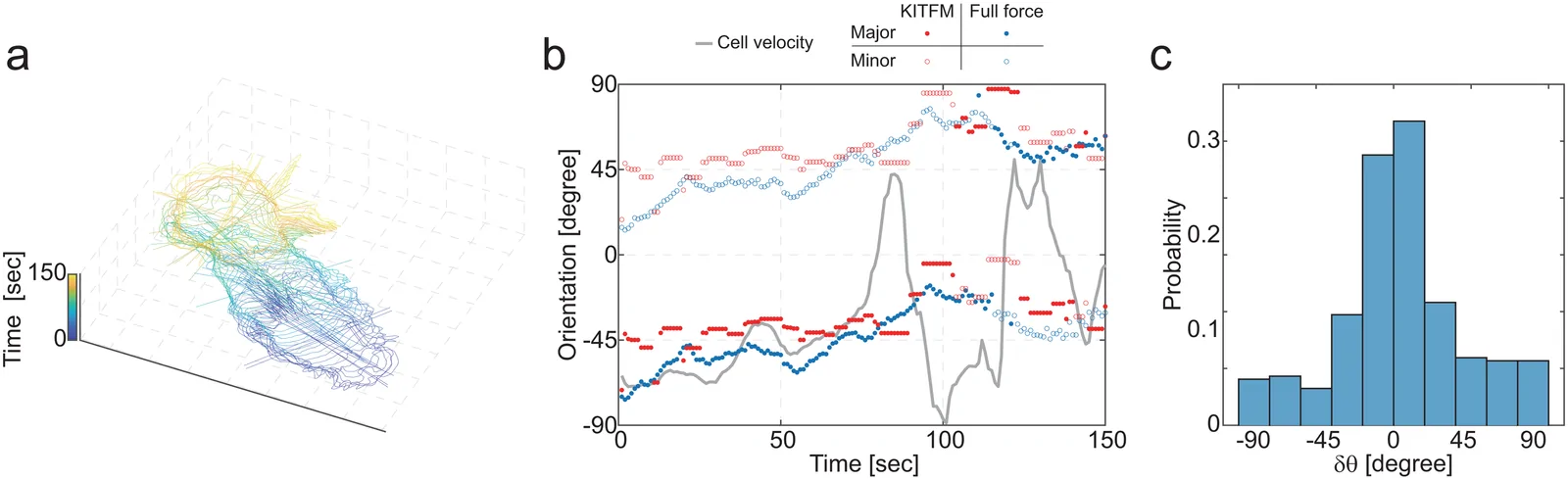
Direct inference of traction dipole. **(a)** Traction force dipole directly inferred using KITFM framework. Lines indicate the major axis of directly inferred traction dipole, which is overlaid on the cell outlines. Time is color-coded. **(b)** Orientational dynamics of force dipole and cell velocity. The orientation of principal axis of traction dipole, determined either using KITFM framework or using full force field, is plotted as a function of time. The orientation of cell velocity is also plotted. **(c)** Correlation between cell velocity and directly inferred dipole orientations. The probability distribution function of orientational difference between cell velocity and directly inferred dipole axis is shown.

## Discussion

In this study, we introduced a novel approach for solving the inverse problem of TFM. Although a number of methods based on spatial information have been developed, temporal information about the system has rarely been utilized for the inversion. The introduced method, termed KITFM, employs Kalman filter algorithm to utilize temporal information for the force inference. We demonstrated that KITFM effectively filters out noise in the temporal domain while preserving fine temporal characteristics. These particular features would be beneficial for studying dynamic cellular forces.

In KITFM one empirical parameter, system noise *Q*, must be set. Because the proposed framework adopts persistent model, it follows force evolution by random walk with a diffusion constant *Q*/2. Therefore, the order of optimal *Q* can be assessed as $Q\sim (\Delta T{)}^{2}/\Delta t$, where Δt is the measurement interval and ΔT is an average change in cellular forces during Δt. Because the inference is not very sensitive to the choice of *Q*, a rough estimation of ΔT is sufficient for determining an appropriate *Q*. One way to achieve this is to compute the force field of two frames close to each other using static methods such as L2-regularization and use the result to determine ΔT and hence *Q*.

Since KITFM is a filter in temporal domain, its accuracy depends on the temporal resolution of measurement. We observed that Δt=1 sec is sufficient to reliably infer traction force of rapidly crawling cells, and the temporal resolution can be reduced to at least Δt~10 sec. This time interval enables ones to simultaneously perform other measurements such as multi-color fluorescence imaging in future extensions. Therefore temporal resolution of the measurement would not be a strong constraint in the implementation of KITFM.

In addition to noise reduction in the temporal domain, the KITFM framework offers several advantages. Because the framework is essentially a sequential Bayesian estimation, prior knowledge about the system can be naturally incorporated into the inference. We provided a simple example in which the cell shape is used to constrain the force field. It is also straightforward to directly determine low-dimensional characteristics without inferring the full force field. Given these particular merits of data assimilation approach, further extensions of KITFM to include other biological information to infer different characteristics would be feasible.

Perhaps the largest bottleneck of proposed framework is that it adopts persistent model and ignores trend in cell dynamics. In practice, this bottleneck can be circumvented by simply increasing the sampling frequency as demonstrated, which is feasible in standard TFM experiments that observe the bright fluorescent signal of fiducial markers. However, it would be still interesting to improve KITFM to precisely match the model to data, because obtaining an accurate model for time-evolution is nearly equal to understanding the phenomenon. Combining the proposed Kalman inversion-based framework with model-independent algorithms [52,53] would be an interesting direction for future research.

## Supporting information

S1 Appendix A PDF file containing Figs A-C. (PDF)
