# Supplementary material for: Traction force microscopy using temporal information
Source: PLoS Comput Biol. 2026 Nov 2;22(11):e1014855. doi: 10.1371/journal.pcbi.1014855 (PMC13750821; doi:10.1371/journal.pcbi.1014855)
Supplement: Supplementary file 1 [file pcbi.1014855.s001.pdf]

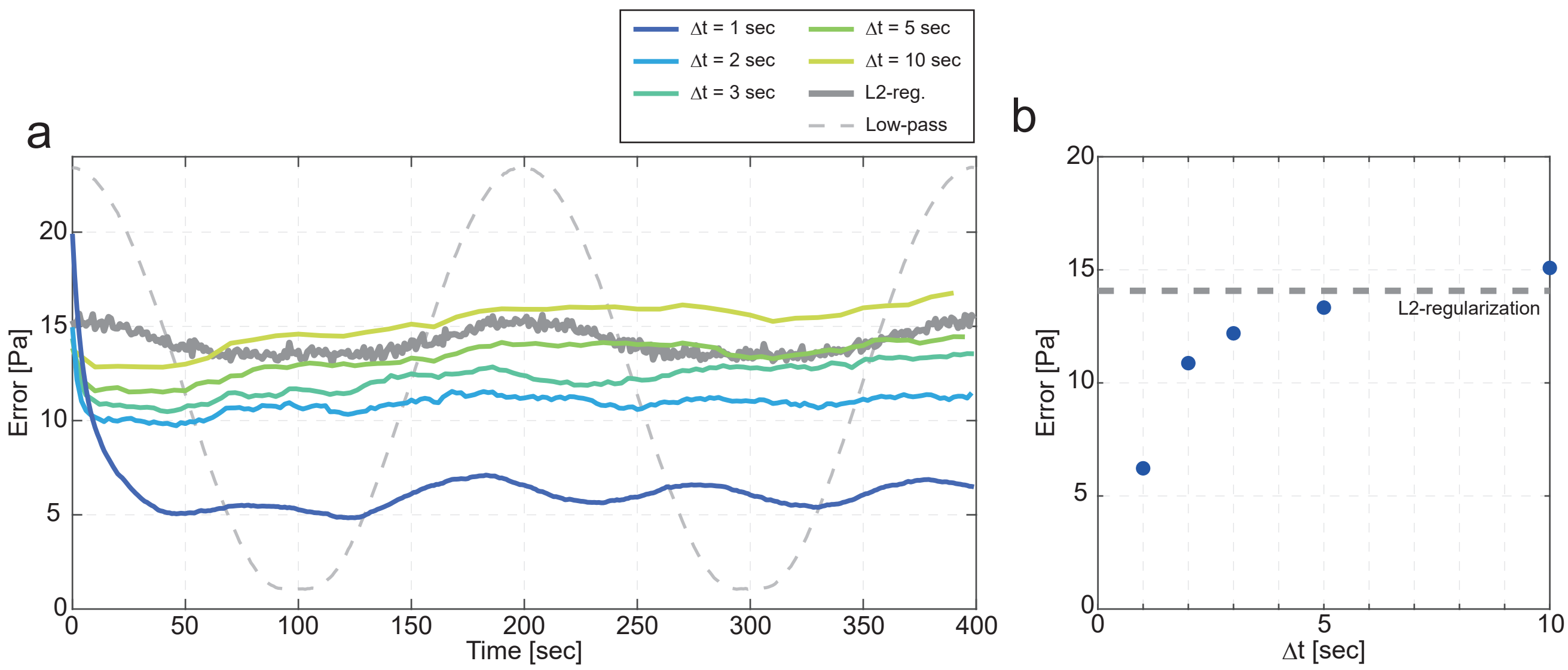

Fig A. Influence of measurement interval on KITFM evaluated using synthetic data, related to Fig 3. (a) Estimation error in KITFM with different measurement interval Δt. Estimation error of KITFM with Δt = 1, 2, 3, 5, and 10 sec is shown as a function of time. Estimation error of L2-regularization and low-pass filtering are also shown. (b) Average estimation error is plotted as a function of Δt. Broken line indicates the error of L2-regularization.

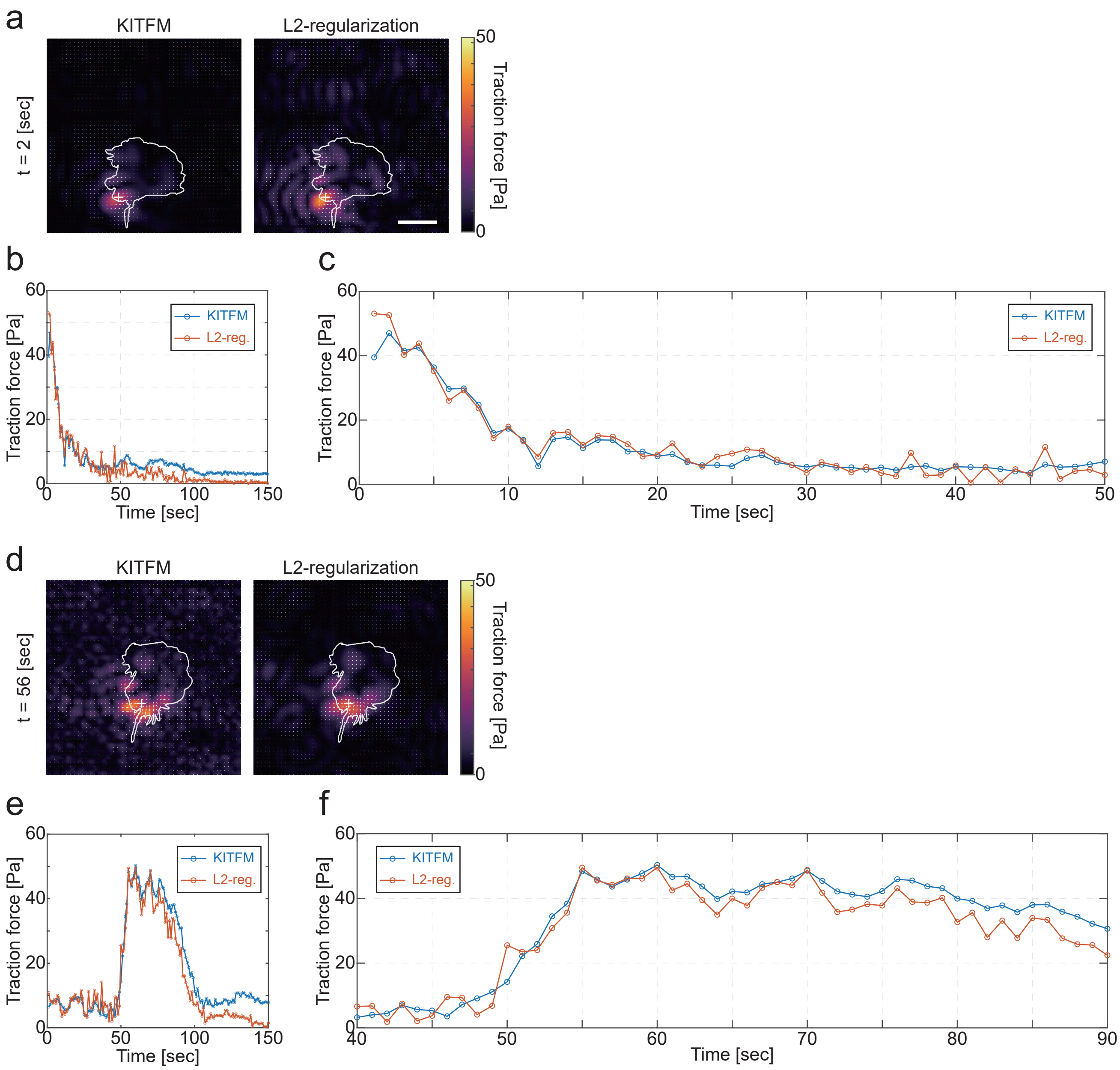

Fig B. Additional analysis comparing KITFM and L2-regularization, related to Fig 4. (a and d) Snapshots of inferred traction forces using KITFM and L2-regularization. The single grids analyzed are shown with + marker. (b and e) The traction force at the grid is plotted as a function of time. (c and f) Enlarged plot of (b) and (e), respectively. (a-c) An example where the traction force takes its maximum value at the beginning of the measurement. (d-f) An example where the traction force takes its maximum value in the middle of the measurement. Scale bar, 10 μm.

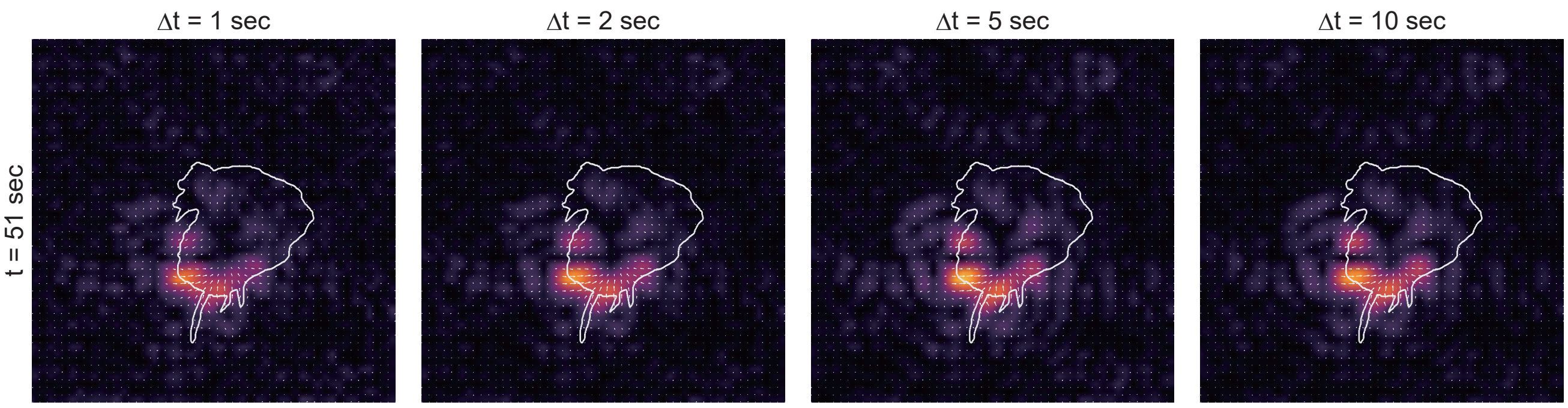

Fig C. Influence of measurement interval on KITFM, related to Fig 4. Force inference using KITFM with different measurement time interval Δt. The influence of measurement time interval on KITFM was evaluated by skipping frames of the same time-sequence used in Fig 4. Inferred force field at t = 51 sec is shown for the measurement interval Δt = 1, 2, 5, and 10 sec.
